# Regenerative medicine: are we there yet?

**DOI:** 10.1038/s41536-016-0005-9

**Published:** 2017-01-05

**Authors:** Stephen Badylak, Nadia Rosenthal

**Affiliations:** 1 McGowan Institute for Regenerative Medicine, Pittsburgh, PA USA; 2 The Jackson Laboratory, Bar Harbor, ME USA; 3 National Heart and Lung Institute, Imperial College London, UK

Despite almost 2500 clinical trials involving stem cells, there is not a single clinical problem that is routinely treated with stem cell therapy because it is better than the standard of care. For all the progress we have made in the fast moving field of regenerative medicine, the capacity to routinely restore functional tissue following traumatic injury or degenerative disease is still beyond reach.

Have the technologies that we now have at hand, such as 3-D printing, deep genomic sequencing and manipulation, or directed stem cell differentiation in the dish, outpaced our understanding of the signals that orchestrate natural development? Should we attempt to restore the epimorphic regeneration potential of humans, releasing the newt within us? Is it even possible to restore a non-blastemal regeneration response in organs like the human brain? It is tempting to extrapolate from the remarkable regenerative capacity of the mammalian neonate and envision the engineering of a similar response in the adult. Or perhaps we should be satisfied with moving the needle from the default inflammation/scar tissue formation response toward a more constructive and functional response, and leave true regeneration to “more primitive” species such as the newt until we understand the upstream signals that regulate these processes.

To advance the pace of progress, scientists are now adopting multidisciplinary strategies, combining evolutionarily conserved principles of developmental biology, immunology, stem cell biology, tissue engineering, biomechanics, and medicine in *ex vivo* and *in vivo* approaches. Recreating the supportive micro-environment of a regenerating zebrafish heart in a patient will require a more profound understanding of the 30% differences in protein coding genes between fish and man, and dynamic cellular processes that rapidly modulate immune response to bypass the physical blockade of infarct scar, promote revascularization to prevent more cell death, and provide the necessary extracellular milieu for restoration of functional cardiac contractile tissue. We’re not there yet, but there’s promising light on the horizon.

